# Deuterium metabolic imaging beyond the brain: mapping tissue metabolism across the body

**DOI:** 10.1007/s10334-026-01327-w

**Published:** 2026-02-28

**Authors:** Maaike M. Konig, Jeanine J. Prompers

**Affiliations:** 1 Imaging and Oncology, University Medical Center Utrecht, Utrecht, The Netherlands; 2 Department of Human Biology, NUTRIM Institute of Nutrition and Translational Research in Metabolism, Maastricht University Medical Center+, Universiteitssingel 50, 6229 ER Maastricht, The Netherlands; 3 Department of Radiology and Nuclear Medicine, Maastricht University Medical Center+, Maastricht, The Netherlands

**Keywords:** Deuterium metabolic imaging, Deuterium magnetic resonance spectroscopy, Body, Liver, Tumor, Metabolism, Glucose, Warburg effect

## Abstract

Deuterium metabolic imaging (DMI) is an emerging magnetic resonance technique that enables non-invasive investigation of in vivo metabolism without the use of ionizing radiation. By administering various deuterium-labeled substrates, different metabolic pathways and fluxes can be probed. To date, most DMI studies have focused on cerebral metabolism; however, its application is rapidly expanding to include metabolic processes in other body organs and tissues, as well as non-brain tumors. This review summarizes the current state of in vivo DMI research beyond the brain, covering studies of the liver, non-brain tumors, and other organs, such as pancreas, kidney, and heart. With ongoing methodological developments and increasing emphasis on clinical translation, DMI holds considerable promise as a versatile tool for studying human metabolism and for future clinical implementation.

## Introduction

The use of stable isotope-enriched substrates in magnetic resonance (MR) provides a safe and non-radioactive manner to study metabolic pathways and fluxes in vivo. A traditionally used technique is to administer carbon-13 (^13^C) labeled substrates and dynamically track the administered substrate and its downstream products using ^13^C-MR spectroscopy (MRS). However, a disadvantage of using ^13^C-MRS is the limited MR sensitivity, caused by the low gyromagnetic ratio and the relatively long T_1_ relaxation times of ^13^C. The limited sensitivity restricts spatial resolution, making it primarily limited to non-localized MRS or single voxel spectroscopy, with a low amount of spatial information [1, 2].

Similar to ^13^C-MRS in combination with ^13^C-labeled substrates, deuterium (^2^H or D) MRS upon administration of ^2^H-labeled substrates can be used to study in vivo metabolism. The wide range of possible applications spurred considerable interest in the 1980s and 1990s, with research focusing on for example body iron storage, lipid metabolism, retinal glucose metabolism, and breast cancer metabolism [3–6]. After this wave of activity, research on ^2^H-MR declined, but it has seen a strong revival in recent years [7, 8].

This recent revival was driven by the publication of two landmark articles about ^2^H-MRS. Lu et al. [7] presented an approach for the quantitative assessment of brain glucose metabolic rates in rats using in vivo ^2^H-MRS upon infusion of [6,6’-^2^H_2_]glucose. De Feyter et al. [8] introduced 3D ^2^H-MRSI, enabling spatially resolved in vivo studies of glucose metabolism and referred to as “Deuterium Metabolic Imaging” (DMI), which they demonstrated in the brain and liver of both rats and human subjects.

^2^H offers several unique characteristics relevant for MR. First, the natural abundance of ^2^H is very low (0.0156%) [9], which in vivo at natural abundance only results in a detectable HDO peak and, depending on the fat content in the region-of-interest, possibly a lipid peak. This simplifies acquisition as it eliminates the need for water and/or fat suppression. It also simplifies quantification as the naturally abundant HDO peak (at baseline) can be used as an internal standard for concentration calculations of the administered ^2^H-labeled substrates and their metabolic products. Second, while ^2^H has a low gyromagnetic ratio (6.54 MHz/Tesla) and therefore a low intrinsic sensitivity, its quadrupolar moment comes with a short T_1_ relaxation time, allowing rapid averaging and spatial (phase) encoding in 2D or 3D, resulting in the gain in sensitivity required for resolving spatial information. The quadrupolar moment also comes with a short T_2_ relaxation time, which leads to broad linewidths. This can create challenges when resolving different compounds in the spectra, but since the ^2^H spectra are sparse, this is in general not a problem for ^2^H-MRS [10].

In addition, the feasibility of spatially resolved ^2^H-MRS has increased with the broader availability of ultra-high field MR scanners. Ultra-high field greatly boosts the sensitivity of ^2^H-MRSI, as the signal-to-noise ratio (SNR) scales supra-linearly with magnetic field strength, while spectral linewidths are largely unaffected [11]. Combined with the wide range of ^2^H-labeled substrates that can be administered, the favorable characteristics of ^2^H make ^2^H-MRSI a powerful and versatile approach for studying metabolic pathways and fluxes in vivo.

A large part of the research since 2017 has focused on cerebral applications of ^2^H-MRS [12–15]. Even though non-cerebral applications of ^2^H-MRS, with investigations of non-brain tumors and healthy body organs like liver, heart and kidneys, are rapidly emerging, these studies have not yet been comprehensively reviewed. This review aims to fill that gap by providing an overview of current ^2^H-MR body applications, highlighting different organs and tissues of interest, different deuterated substrates and metabolic pathways, methodological advances, and translational opportunities and challenges.

## ^2^H-MRS applications across organs and tissues in the body

This paragraph provides an overview of current ^2^H-MRS applications across organs and tissues in the body, focusing on the deuterated substrates used, their metabolic fate in different organs and conditions, and methodological approaches. To clarify substrate-specific pathways and to facilitate comparison across studies, this information is summarized in Tables 1 and 2, respectively.

**Table 1:** Deuterated compounds which have been used for ^2^H MRS in body applications

Substrate	Metabolic pathway	Potentially detectable downstream products*
[6,6’-^2^H_2_]glucose or [^2^H_7_]glucose	Anaerobic glycolysis	[3,3’-^2^H_2_]lactate HDO
	TCA cycle	[4,4′-^2^H_2_]Glx HDO
	Glycogenesis	None (^2^H-glycogen is NMR invisible in vivo)
3-O-C^2^H_3_-glucose	Cannot be metabolized	None
[3,3’,3″-^2^H_3_]pyruvate	Anaerobic glycolysis	[3,3’-^2^H_2_]lactate HDO
	TCA cycle	[4,4′-^2^H_2_]Glx HDO
[6,6’-^2^H_2_]fructose	Fructolysis → gluconeogenesis	[6,6’-^2^H_2_]glucose
	Fructolysis → glycolysis	[3,3’-^2^H_2_]lactate HDO
	Fructolysis → TCA cycle	[4,4′-^2^H_2_]Glx HDO
[2,2’,2″-^2^H_3_]acetate	TCA cycle	[4,4′-^2^H_2_]Glx HDO
[^2^H_31_]palmitic acid or [^2^H_15_]octanoic acid	β-oxidation → TCA cycle	[4,4′-^2^H_2_]Glx HDO
	β-oxidation → ketogenesis	^2^H-3-hydroxybutyrate HDO
^2^H_2_O	De novo lipogenesis (only in liver)	^2^H-lipids
[^2^H_9_]TMA	TMA oxidation (only in liver)	[^2^H_9_]TMAO
[^2^H_9_]choline	Kennedy pathway	^2^H-choline-containing species
	Choline oxidation	[^2^H_9_]betaine HDO
[2,3-^2^H_2_]fumarate	TCA cycle / hydration of fumarate	[2,3-^2^H_2_]malate HDO
PAMAM-G5-[^2^H_3_]Ac	Is not metabolized, but cleared by kidneys	None

*For pathways in which deuterium can be lost to water with formation of HDO, the potential formation of HDO is indicated in the table. For these pathways, only the downstream metabolites with full retention of the ^2^H label are shown, and the metabolites with partial label loss are omitted

**Table 2:** Overview of ^2^H-MRS applications across organs and tissues in the body

Organ/Tissue	Species	Condition	Substrate	Field strength	Coil	Sequence*	Key finding(s)	Ref
Liver	Rat and human	Healthy	[6,6’-^2^H_2_]glucose	4 T (human) & 11.7 T (rat)	Surface coil (human) & volume coil (rat)	3D MRSI	Peak at 3.8 ppm attributed to ^2^H-glucose/^2^H-glycogen; no downstream Glx or lactate labeling in liver	[8]
	Rat and mouse	Healthy	[6,6’-^2^H_2_]glucose	11.7 T	Volume coil	Non-localized MRS & 3D MRSI (rat) and NMR (mouse liver extract)	^2^H-glycogen is MR invisible in vivo; intraperitoneal administration leads to higher glucose signals in the liver compared to intravenous administration	[24]
	Human	Healthy	[6,6’-^2^H_2_]glucose	7 T	Body array coil	3D MRSI	Maximum glucose signal 60–100 min after oral intake; large field of view allows simultaneous assessment of gastric emptying, and hepatic and renal glucose uptake	[25]
	Human	Healthy	[6,6’-^2^H_2_]glucose	7 T	Triple-tuned surface coil	3D MRSI	Maximum glucose signal 60–100 min after oral intake; interleaved measurements of liver glucose uptake (DMI) and glycogen storage (^13^C-MRS)	[26]
	Human	Healthy	[6,6’-^2^H_2_]glucose	3 T	Surface coil	Non-localized MRS & 3D MRSI	DMI of the abdomen is feasible at clinical field strength; glucose dose can be reduced to 0.5 g/kg	[28]
	Human	Gastric bypass	[6,6’-^2^H_2_]glucose	7 T	Surface coil	3D MRSI	Faster hepatic glucose kinetics in patients with gastric bypass	[29, 31]
	Mouse	Healthy	[6,6’-^2^H_2_]fructose and [6,6’-^2^H_2_]glucose	11.7 T	Surface coil	3D MRSI	Hepatic uptake and turnover of fructose higher than for glucose	[33]
	Rat	Fatty liver	[2,2’,2’’-^2^H_3_]Na-acetate	9.4 T	Surface coil	Non-localized MRS & 3D MRSI	^2^H-Glx detected; no significant differences in uptake or metabolism of acetate in rats with fatty livers compared to controls	[34]
	Rat	Fatty liver	[^2^H_31_]palmitic acid and [6,6’-^2^H_2_]glucose	9.4 T	Surface coil	Non-localized MRS & 3D MRSI	No significant differences in uptake or metabolism of palmitic acid or glucose in rats with fatty livers compared to controls	[35]
	Mouse	Fatty liver	[^2^H_15_]octanoic acid	11.1 T	Saddle coil	FLASH and two-point Dixon	β-oxidation declined more strongly in mice with fatty livers compared to controls	[36]
	Rats	Healthy	^2^H_2_O	11.7 T	Volume coil	STEAM (+ CHESS water suppression to acquire lipid signals)	In vivo ^2^H-lipid levels correlated with ex vivo measurement of hepatic DNL	[37]
	Human	Healthy	^2^H_2_O	7 T	Surface coil	3D MRSI	South Asian men had an increased Fraction of liver fat originating from DNL compared with white European men	[38]
	Mouse	Healthy	[^2^H_9_]TMA	15.2 T	Surface coil	Non-localized MRS & 2D MRSI	In females, TMAO formation in the liver and bladder was measured, but not in males	[39]
(Non-brain) tumors	Mouse	Lymphoma	[6,6’-^2^H_2_]glucose	9.4 T	Surface coil	Slice-selective MRS & 3D MRSI	Maximum tumor glycolytic flux of 990 µM/min; flux decreased 48 h after treatment	[47]
	Mouse	Pancreatic cancer	[6,6’-^2^H_2_]glucose	15.2 T	Surface coil	Non-localized MRS & 2D MRSI	Much faster glucose build-up and slower rate of lactate production than [46], resulting in a glycolytic flux of only 6 µM/min	[48]
	Mouse	Renal carcinoma	[6,6’-^2^H_2_]glucose	11.7 T	Surface coil	Non-localized MRS & 3D MRSI	Similar glucose time curves, but highest lactate concentration twice as low as in [46], possibly due to lower glucose dose and/or more efficient lactate removal by renal tumors	[49]
	Mouse	Advanced melanoma	[^2^H_7_]glucose and [6,6’-^2^H_2_]glucose	11.1 T	Half-saddle coil	2D FLASH, non-localized MRS & slice-selective MRS	Increased labeling of HDO pool with [^2^H_7_]glucose than with [6,6’-^2^H_2_]glucose	[50]
	Rat	Breast cancer bone metastases	3-O-C^2^H_3_-glucose (OMG)	7 T	Surface coil	Non-localized MRS & 2D MRSI	OMG resonance (at 3.5 ppm) is better separated from HDO than [6,6’-^2^H_2_]glucose	[53]
	Mouse	Pancreatic cancer	[3,3’,3’’-^2^H_3_]pyruvate and [6,6’-^2^H_2_]glucose	15.2 T	Surface coil	Non-localized MRS & 2D MRSI-SSFP	Unlike for glucose, lactate production from pyruvate was largely non-tumor-specific	[54]
	Mouse	Liver cancer	[6,6’-^2^H_2_]fructose and [6,6’-^2^H_2_]glucose	9.4 T	Surface coil	2D MRSI	Lactate production rate two-fold lower for fructose than for glucose, but HDO production rate comparable	[55]
	Mouse	Renal carcinoma	[^2^H_9_]choline and [6,6’-^2^H_2_]glucose	11.7 T	Surface coil	Non-localized MRS & 3D MRSI	DMI allows simultaneous, distinguishable detection of choline and glucose uptake and metabolism	[49]
	Mouse	Lymphoma, colorectal carcinoma and breast cancer	[2,3-^2^H_2_]fumarate	7 T	Surface coil	Non-localized MRS & 3D MRSI	Malate/fumarate ratio increased significantly post-treatment	[56]
	Mouse	Lymphoma	[2,3-^2^H_2_]fumarate	7 T	Surface coil	Non-localized MRS & 3D MRSI	Oral dosing of fumarate yielded comparable results to intravenous administration	[57]
	Mouse	Breast cancer	[2,3-^2^H_2_]fumarate	7 T	Surface coil	Non-localized MRS	Non-toxic, lower doses of fumarate remain sufficiently sensitive to detect tumor cell death	[58]
	Mouse	Pancreatic carcinoma and colorectal carcinoma	^2^H_2_O	9.4 T & 11.7 T	Volume coil	2D MRSI	Higher ^2^H labeling of tumor compared to healthy tissue; strong tumor contrast after one week of labeling	[59]
	Mouse	Pancreatic carcinoma and colorectal carcinoma	^2^H_2_O	1.5 T	Volume coil	2D FLASH	Tumor-specific labeling after one day; reduction in tumor ^2^H signal from day 1 after radiotherapy or chemotherapy, preceding morphological changes	[60]
Pancreas	Mouse	Pancreatic cancer versus pancreatitis	[6,6’-^2^H_2_]glucose	15.2 T	Surface coil	2D MRSI-SSFP & 2D multi-echo SSFP	DMI detects lactate labeling in pancreatic tumors but not in pancreatitis, allowing differentiation of the two	[64]
Kidney	Human	Healthy	[6,6’-^2^H_2_]glucose	7 T	Body array coil	3D MRSI	Renal glucose uptake kinetics resembles that in liver	[25]
	Human	Healthy	[6,6’-^2^H_2_]glucose and ^2^H_2_O	7 T	Body array coil	3D concentric ring trajectory MRSI	Renal glucose uptake kinetics resembles interstitial glucose level dynamics; measurement of ^2^H_2_O uptake and distribution	[65]
	Human	Oncocytoma (benign renal tumor)	^2^H_2_O	3 T	Surface coil	Non-localized MRS & 3D MRSI	Higher HDO labeling in oncocytoma compared to normal-appearing kidney tissue	[66]
	Mouse	Healthy	PAMAM-G5-[^2^H_3_]Ac	15.2 T	Surface coil	2D MRSI-SSFP	Visualization of renal uptake and clearance, and intrarenal distribution	[67]
Heart	Mouse	Healthy	[2,2′,2″-^2^H_3_]acetate and [6,6’-^2^H_2_]glucose	16.4 T	Surface coil	3D MRSI	Higher HDO accumulation for acetate compared to glucose; ^2^H-Glx peak visible after acetate administration but not after glucose administration	[69]
Skeletal muscle	Human	Healthy	Endogenous HDO	7 T	Body array coil	3D MRSI	Orientation-dependent residual quadrupolar couplings	[71]
Fetoplacental tissue	Mouse	Preeclampsia	[6,6’-^2^H_2_]glucose	15.2 T	Surface coil	Non-localized MRS & 2D MRSI	Increased glucose, HDO, and especially lactate labeling of fetuses and placenta of preeclamptic mice compared with healthy controls	[73]
Brown adipose tissue	Rat	Healthy	[6,6’-^2^H_2_]glucose	9.4 T	Surface coil	2D MRSI	Brown adipose tissue of cold-acclimatized rats showed higher glucose uptake, together with higher HDO and lower ^2^H-Glx labeling compared with controls, suggesting a shift toward anaerobic glycolysis	[77]
Inflammation	Mouse	Inflammation	[6,6’-^2^H_2_]glucose	9.4 T	Surface coil	Non-localized MRS	Inflammatory sites showed faster glucose decline, higher lactate labeling, and a smaller initial HDO rise, indicating enhanced anaerobic glycolysis	[81]
	Mouse	Inflammation	PAMAM-G5-[^2^H_3_]Ac	15.2 T	Surface coil	2D MRSI-SSFP	Nanoparticles accumulated in lymph nodes of the inflamed leg and not in the contralateral healthy leg	[67]

*MRSI without further specifications refers to phase-encoded free induction decay MRSI

### Liver

The liver plays a key role in a multitude of metabolic processes, including the maintenance of glucose homeostasis. It acts as a reserve for systemic glucose. When blood glucose levels are high, the pancreas secretes insulin, which promotes hepatic glucose storage. In fact, hepatic glucose uptake accounts for ~25% of whole-body uptake in the postprandial state [16]. This glucose is stored in the form of glycogen. When blood glucose levels are low, the pancreas secretes glucagon, which promotes the conversion of glycogen back to glucose [17]. Additionally, the liver plays a key role in de novo lipogenesis (DNL), a metabolic pathway in which fatty acids are produced from carbohydrates.

These metabolic processes in the liver are disturbed in several pathological conditions associated with metabolic dysfunction. For example, in type 1 diabetes, the pancreas produces little to no insulin, leading to a dysregulation of glucose storage in the liver and contributing to hyperglycemia [18]. In type 2 diabetes, which is closely associated with metabolic syndrome and obesity, insulin resistance occurs in the liver and skeletal muscle, and the pancreas secretes insufficient insulin, also causing hyperglycemia [19]. Metabolic dysfunction-associated steatotic liver disease (MASLD), which also often develops in the context of metabolic syndrome and obesity, is currently the most widespread chronic liver disease, affecting almost 40% of adults [20]. MASLD is characterized by the accumulation of lipids in the liver plus at least one cardiometabolic risk factor [21]. The accumulation of lipids in MASLD is associated with an increased influx of fatty acids from the circulation, dysregulated fatty acid β-oxidation, and elevated DNL [22]. Elevated DNL has also been related to high consumption of fructose, which is a potent stimulator of DNL [23].

Currently, non-invasive and radiation-free methods to assess carbohydrate and lipid metabolism directly in the liver are limited, and current knowledge about these processes is often based on indirect measurements, for example, in plasma. Against this background, DMI emerges as a promising tool to gain new insights into hepatic metabolism in both healthy and disease conditions.

#### Glucose

In addition to providing the first demonstration of DMI in the brain, De Feyter *et al.* [8] also applied the technique in both rat and human liver in the same paper. One to two hours after intravenous (rat) or oral (human) [6,6’-^2^H_2_]glucose administration, the ^2^H-MR spectra of the liver clearly showed a second peak besides the HDO peak, which was attributed to the combined signal of ^2^H-glucose and ^2^H-glycogen. However, unlike in the brain, labeling of downstream metabolites, such as glutamate and glutamine (combined referred to as Glx) and lactate, was not observed in the liver. To be able to quantify the contributions of ^2^H-glucose versus ^2^H-glycogen to the observed signal at 3.8 ppm, they conducted another study investigating the MR-visibility of ^2^H-glycogen [24]. Nuclear Magnetic Resonance (NMR) analysis of isolated liver glycogen of mice fed with [6,6’-^2^H_2_]glucose showed that the T_2_ of less than 2 ms of ^2^H-glycogen makes it practically NMR invisible. Thus, the peak measured at 3.8 ppm in the liver in vivo can be completely attributed to ^2^H-glucose.

The same study explored differences in hepatic glucose dynamics between intravenous and intraperitoneal infusion. During two hours, rats were infused either intravenously or intraperitoneally with 1.5 g/kg body weight [6,6’-^2^H_2_]glucose while simultaneously acquiring DMI scans of their livers at 11.7 Tesla (T). For intravenous administration, the glucose signal plateaued around the HDO level, while for intraperitoneal administration, the glucose signal continued to increase until the end of the experiment and became around twice as high as the HDO signal. The lower signal during intravenous injection could be explained by ^2^H-glucose uptake in other tissues before reaching the liver, whereas with the intraperitoneal route glucose first passes through the liver via the portal circulation, more closely reflecting physiological postprandial uptake. Additionally, the measured glucose uptake spatially varied within the liver, indicating that DMI may be able to map the vascularization of the liver [24].

**Fig. 1:**
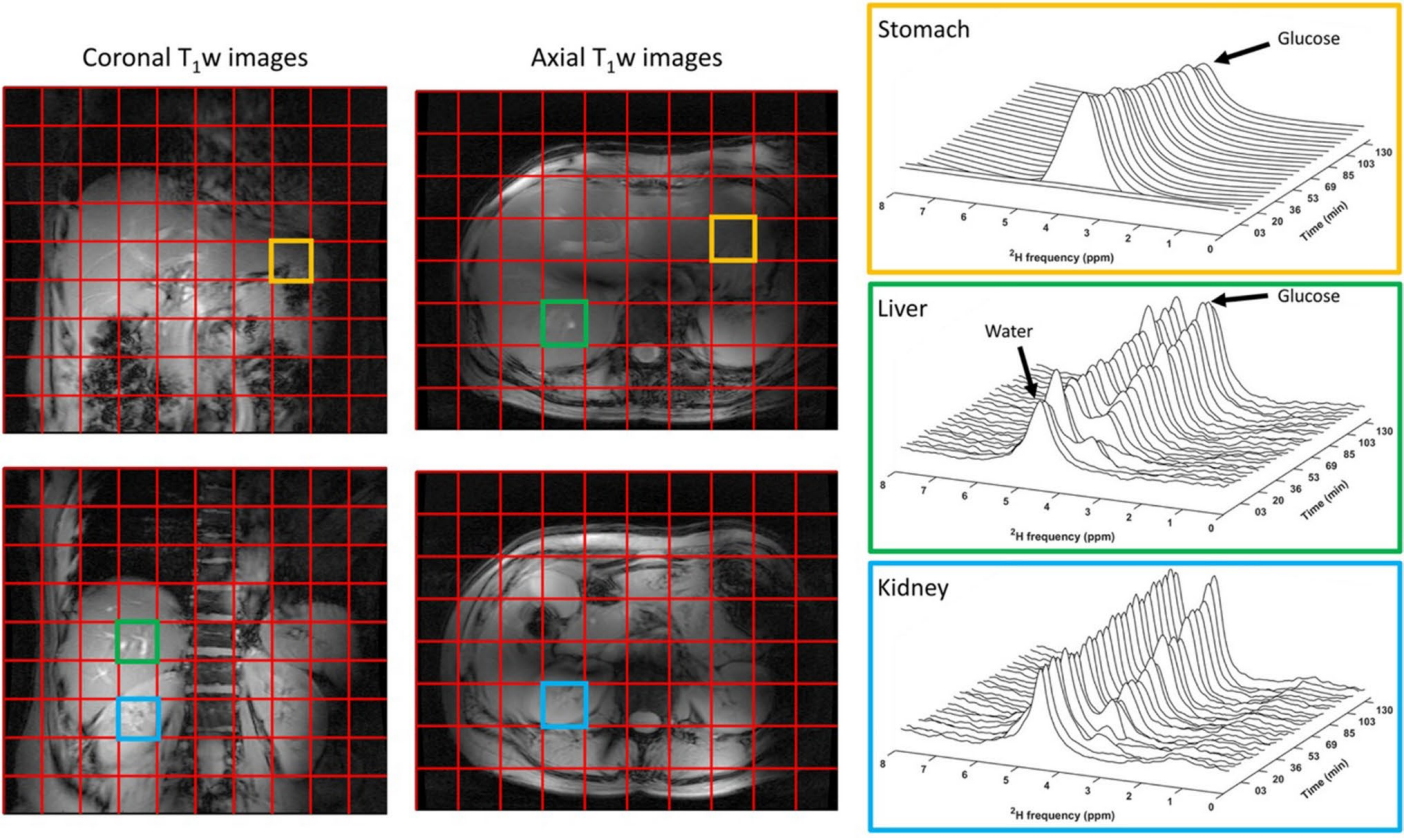
Dynamic 3D DMI data acquired of the human abdomen with a ^2^H body array, at baseline and 0–130 min after oral intake of [6,6’-^2^H_2_]glucose. Left: DMI voxel grid overlaid on coronal and axial T_1_-weighted images. Right: Time-resolved ^2^H spectra from voxels in the stomach (orange), liver (green), and kidney (blue). The large field of view enabled by the ^2^H body array allows simultaneous acquisition of metabolic information from these three organs. This figure was reproduced from Gursan et al. [25] under the Creative Commons CC BY license

Building on these findings, subsequent studies by other groups applied dynamic DMI in the human liver to investigate hepatic glucose handling over time. In two ultra-high field (7 T) studies, subjects received an oral dose of 60 g of [6,6’-^2^H_2_]glucose, and DMI scans were performed up to ~ 3 h after intake, with a temporal resolution of 4 to 10 min, showing a maximum glucose signal in the liver around 60–100 min after intake [25, 26]. In one of those studies, a triple-channel ^1^H/^2^H/^13^C radiofrequency (RF) coil was employed, allowing interleaved measurements of liver glucose uptake with DMI and liver glycogen storage with ^13^C-MRS [26]. In the other study, a ^2^H body array was developed, enabling a large field of view and thereby allowing the simultaneous assessment of gastric emptying and hepatic (and renal) glucose uptake by DMI (Fig. 1) [25]. This is particularly relevant for metabolic diseases such as type 2 diabetes, in which gastric emptying abnormalities are common [27]. To translate this method to clinical field strength, Wodtke et al*.* [28] performed a similar study at 3 T. They showed that DMI of the abdomen is also feasible at lower field strength and obtained dynamic DMI data from stomach, duodenum, liver, and kidney. In most (early) in vivo DMI studies in humans, including brain studies, a dose of 0.75 g/kg body weight [6,6’-^2^H_2_]glucose has been used. Wodtke et al*.* [28] tested different doses of [6,6’-^2^H_2_]glucose and concluded that for abdominal applications of DMI at 3 T, the dose could be reduced to 0.5 g/kg body weight without compromising spectral quality or reliability of quantification.

Poli et al*.* [29] used DMI to investigate the differences in postprandial hepatic glucose metabolism between patients who underwent bariatric surgery and healthy controls. They specifically studied patients who had undergone a Roux-en-Y gastric bypass (RYGB), a procedure that bypasses most of the stomach and the duodenum, directing ingested food straight into the jejunum. This results in higher postprandial plasma glucose levels, stimulating insulin secretion. When the insulin response is excessive, plasma glucose levels may drop too low, which is called post-bariatric hypoglycemia, a condition that occurs in a small group of RYGB patients [30]. Since the liver plays such an important role in maintaining glucose homeostasis, Poli et al*.* hypothesized that the altered plasma glucose and insulin levels in RYGB patients are associated with altered hepatic glucose handling. Ten RYGB patients with mild post-bariatric hypoglycemia and ten healthy controls underwent dynamic DMI of the liver over the course of 2.5 h after oral administration of 60 g of [6,6’-^2^H_2_]glucose. In the RYGB patients, a sharp increase in hepatic ^2^H-glucose was observed, followed by a decrease, whereas healthy controls showed a more gradual increase and subsequent stabilization. Together with plasma concentrations of glucose and insulin and deuterium enrichment of plasma glucose, the DMI data were employed to create a mathematical model of postprandial hepatic glucose kinetics [31]. Modeling results indicated that, after 150 min, nearly all ingested glucose had reached the liver in RYGB patients but not in healthy controls, yet total hepatic disposal and first-pass extraction were comparable between the two groups.

#### Fructose

Unlike glucose, hepatic uptake of fructose is largely independent of the liver’s energy status, and most fructose delivered through the portal vein is rapidly extracted by the liver [32]. Hendriks et al. [33] aimed to show the feasibility of using DMI in combination with intravenous administration of [6,6’-^2^H_2_]fructose (main resonance at 3.7 ppm) to measure uptake and metabolism of fructose in the mouse liver and compare these results to the uptake and metabolism of [6,6’-^2^H_2_]glucose. After bolus injection of the substrate, a more than two-fold higher initial uptake and faster decay was observed with fructose compared to glucose. With slow infusion of the substrate, lower signals were measured with fructose compared to glucose. Lastly, for both protocols, labeling of the water pool with deuterium was faster after fructose administration as compared to glucose. These results support the notion that fructose undergoes more extensive extraction and faster metabolic turnover in the liver than glucose.

#### Acetate

Because acetate feeds directly into the tricarboxylic acid (TCA) cycle, DMI with acetate may enable assessment of hepatic TCA cycle flux via Glx labeling, as opposed to glucose, with which Glx labeling is not observed in the liver. Ehret et al. [34] used the substrate [2,2’,2’’-^2^H_3_]Na-acetate to study alterations in TCA cycle flux in diet-induced fatty livers of rats compared to healthy controls. However, acetate administration proved challenging. Bolus injection was not physiologically tolerable, and slow infusion over an extended period (120 min) was necessary, which limited the analysis of downstream products. Nonetheless, ^2^H-Glx was detected in the livers of both healthy rats and rats with diet-induced fatty livers; however, no significant differences in hepatic acetate uptake or metabolic breakdown into Glx were found between the two groups.

#### Fatty acids

In order to study both disturbances in carbohydrate and lipid metabolism in the fatty liver, Ehret et al. [35] applied DMI in combination with a bolus intraperitoneal injection of either [6,6’-^2^H_2_]glucose or [^2^H_31_]palmitic acid (a deuterated fatty acid) in healthy rats and rats with diet-induced fatty livers. There were no significant differences in hepatic glucose or palmitic acid uptake and metabolism between the two groups although the hepatic concentration of deuterated palmitic acid seemed to be higher in the rats with fatty livers. The lack of significant results was explained by the relatively mild degree of liver impairment in the study population, combined with a suboptimal time scale and small group size.

In addition to [^2^H_31_]palmitic acid, the shorter-chain fatty acid [^2^H_15_]octanoic acid has also been used to assess hepatic fatty acid metabolism with DMI. In a study by McLeod et al. [36], mice were fed either a standard chow diet (healthy controls) or a high-fat diet to induce MASLD. At baseline and after 8.5, 17, 24, and 36 weeks of diet, the animals received an intravenous injection of [^2^H_15_]octanoic acid and were imaged using ^2^H fast low-angle single shot (FLASH) and two-point Dixon sequences. Relative β-oxidation, defined as HDO produced per mg [^2^H_15_]octanoic acid per gram of liver tissue, declined more strongly over the 36-week diet period in the high-fat diet group compared with controls. These findings demonstrate that DMI with deuterated fatty acids can noninvasively capture alterations in hepatic fatty acid β-oxidation associated with MASLD progression.

#### ^2^H_2_O

The liver not only takes up fatty acids from the circulation, but also synthesizes lipids de novo (DNL) from carbohydrates. The gold standard for DNL quantification relies on plasma-based measurements, which are indirect and may underestimate hepatic DNL when newly synthesized fatty acids remain stored within the liver. Although liver biopsy provides a more direct assessment, this invasive approach is not suitable for human studies. Gursan et al. [37] demonstrated the feasibility of directly detecting deuterium labeling in liver lipids in vivo using localized ^2^H-MRS after a week of ^2^H_2_O (heavy water) administration to rats. The in vivo measurements of ^2^H-lipid levels correlated strongly with *ex vivo* NMR estimates of hepatic DNL, supporting the potential of ^2^H-MRS as a quantitative, noninvasive method for assessing hepatic DNL in vivo. Very recently, this approach has also been demonstrated in humans, revealing a higher fraction of DNL-derived liver fat in South Asian participants compared with age- and body mass index-matched European subjects (preprint) [38].

#### Trimethylamine

Recently, Dessau et al. [39] demonstrated the feasibility of using DMI to study hepatic metabolism of trimethylamine (TMA) to trimethylamine *N*-oxide (TMAO), a metabolite linked to cardiovascular, renal, and neurological disorders. Following oral administration of [^2^H_9_]TMA in mice, ^2^H-MR spectra acquired at 15.2 T revealed TMA signals in the stomach and, in females, clear TMAO formation in the liver and bladder, consistent with renal clearance. No hepatic TMAO was detected in males, in line with testosterone-mediated downregulation of the TMA-to-TMAO pathway. This study illustrates how DMI can provide spatially resolved insights into hepatic TMAO formation, although the small chemical shift difference between [^2^H_9_]TMA and [^2^H_9_]TMAO (~0.4 ppm) may limit spectral separation at clinical field strengths [39].

### (Non-brain) tumors

Much of the DMI research to date has been directed toward the characterization of tumor metabolism and the evaluation of tumor treatment response. Tumor metabolism can differ substantially from metabolism in healthy tissues. First, many tumors have an increased uptake of glucose compared to healthy tissue. In addition, glucose is often metabolized in a different manner. In healthy cells, glucose is predominantly oxidized via the TCA cycle and oxidative phosphorylation under aerobic conditions. This pathway leads to the production of Glx. When there is not sufficient oxygen present, healthy cells will switch to anaerobic glycolysis, which leads to the production of lactate. Many tumor cells favor the anaerobic glycolysis pathway regardless of the presence of oxygen, a phenomenon called the Warburg effect [40]. Lactate can promote angiogenesis and activate tumor growth pathways, and may be involved in multiple stages of carcinogenesis [41]. In addition to glucose, fructose metabolism also plays a key role in different types of cancer [42]. Cancer cells require an increased lipid supply to sustain proliferation, and fructose can be used as a precursor for lipid synthesis. Additionally, similar to glucose, the metabolism of fructose in tumor tissue is shifted toward the formation of lactate, supposedly supporting tumor growth [43]. Multiple studies have indicated that increased fructose uptake in tumors is associated with poor prognosis [42]. Lastly, tumor cells frequently exhibit increased choline uptake. Choline is a precursor of cell membrane phospholipids, and elevated choline uptake has been associated with high rates of cell membrane synthesis and cell proliferation [44].

Not only vital tumor tissue exhibits specific metabolic changes, but also necrotic tumor tissue shows altered metabolite conversions due to loss of cellular integrity. In studies using hyperpolarized ^13^C-MRS, it has been shown that the conversion of exogenously supplied fumarate to malate is much faster in necrotic tissue compared to vital tissue [45, 46]. Fumarate and malate are intermediates of the TCA cycle, with fumarase catalyzing the conversion of fumarate to malate. Under normal conditions, access of exogenously supplied fumarate, initially accumulating in the extra-cellular space, to the mitochondrial enzyme fumarase is limited, and the conversion to malate is slow. However, loss of plasma membrane integrity in necrotic cells increases the access of fumarate to fumarase, leading to a more rapid conversion to malate. Therefore, the conversion rate of fumarate to malate can serve as a marker of tumor necrosis in the assessment of therapy response.

#### Glucose

**Fig. 2:**
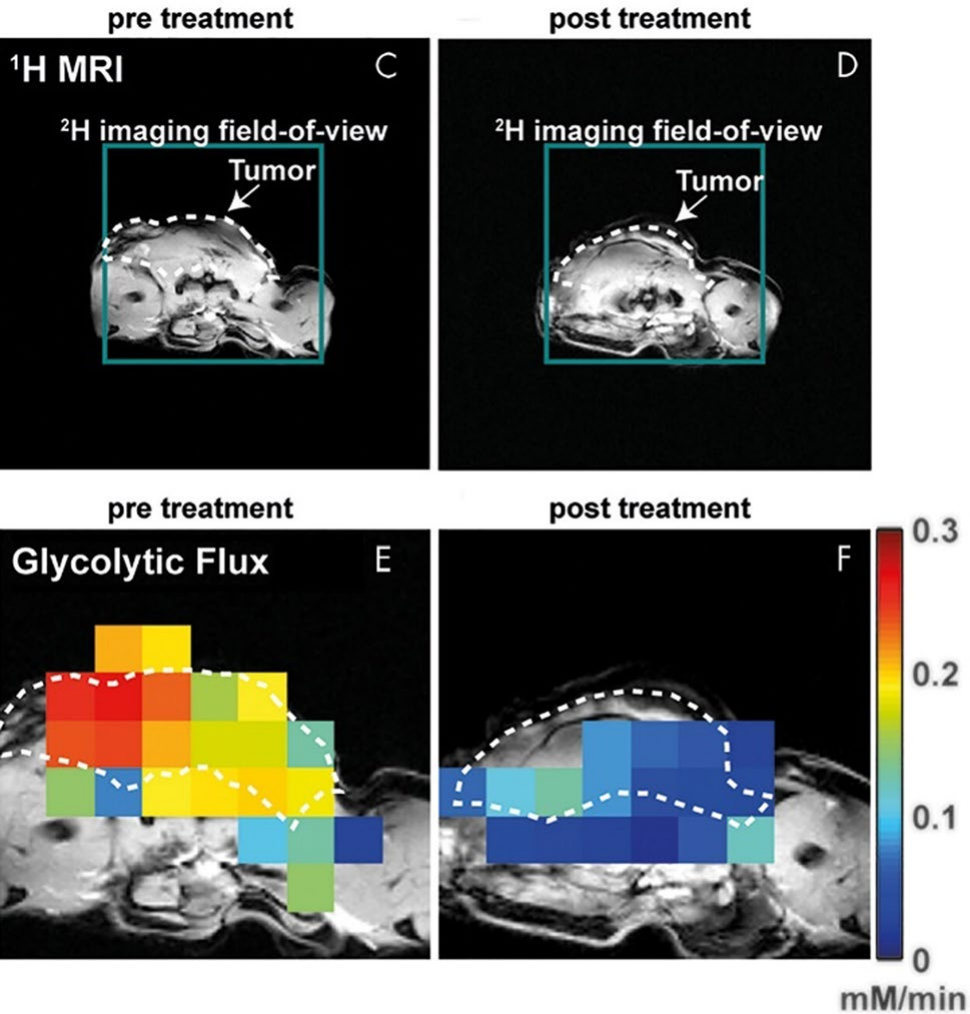
Dynamic DMI data of a murine lymphoma model. Top: DMI field of view overlaid on anatomical axial images with the tumor indicated. Bottom: Glycolytic flux, as determined from the dynamic DMI data, before (left) and after (right) treatment. The glycolytic flux decreased within 48 h after treatment. This figure was reproduced from Kreis et al. [47] under the Creative Commons CC BY license

[6,6’-^2^H_2_]glucose is the most commonly used deuterated glucose substrate in DMI research. When [6,6’-^2^H_2_]glucose enters anaerobic glycolysis, this will lead to the production of [3,3’-^2^H_2_]lactate, enabling the quantification of glycolytic flux. Several studies have applied DMI with intravenous injection of [6,6’-^2^H_2_]glucose in pre-clinical, non-brain tumor models. Using a ^2^H surface transmit–receive coil and non-localized ^2^H-MRS at 9.4 T, Kreis et al. [47] measured a maximum tumor glycolytic flux of 990 *µ*M/min in a subcutaneous murine lymphoma model. Moreover, with dynamic 3D ^2^H-MRSI, it was shown that the glycolytic flux was spatially heterogeneous within the tumor and that the flux decreased already 48 hours after treatment, suggesting potential for early treatment response monitoring (Fig. 2). In orthotopic pancreatic cancer mouse models, dynamic ^2^H non-localized and 2D MRSI measurements at 15.2 T showed a much faster glucose build-up and a slower rate of lactate production compared to the study of Kreis et al. [47], resulting in a glycolytic flux of only 6 *µ*M/min [48]. However, in healthy tissue, no lactate signals were detected at all, thus, the relatively slow conversion rate still distinguished the pancreatic tumor from its surroundings [48]. In mice with subcutaneously grown human renal carcinoma cells, dynamic ^2^H non-localized MRS and 3D MRSI at 11.7 T yielded similar time curves of deuterated glucose in tumor tissue compared to the study of Kreis et al. [47], but the highest measured deuterated lactate concentration was twice as low [49]. The latter could be explained by a lower [6,6’-^2^H_2_]glucose dose (1.3 g/kg in [49] versus 2 g/kg in [47]). Another possible explanation is that renal tumors remove lactate more efficiently. Collectively, these studies demonstrate that DMI with [6,6’-^2^H_2_]glucose reveals pronounced tumor-type specific differences in glucose metabolism, underscoring the importance of metabolic imaging in tumor characterization.

To overcome the limited sensitivity of [6,6’-^2^H_2_]glucose, Chang et al*.* [50] used perdeuterated [^2^H_7_]glucose as substrate in a mouse model of advanced melanoma. Consistent with studies using [6,6’-^2^H_2_]glucose, higher lactate signals were detected in tumor tissue compared to healthy (contralateral) tissue after administration of [^2^H_7_]glucose. In addition, faster labeling of the HDO pool was observed in tumor tissue, providing an even more sensitive marker of glycolytic flux. When equal doses of [^2^H_7_]glucose or [6,6’-^2^H_2_]glucose were administered, the tumor HDO signal measured 6 min post-injection was ~ 2.7-fold higher with [^2^H_7_]glucose, demonstrating its superior sensitivity. However, [^2^H_7_]glucose is more expensive than [6,6’-^2^H_2_]glucose, and future studies investigating optimal dosing and administration strategies for both compounds are needed to balance cost against SNR performance.

3-O-Methylglucose (OMG) is a non-metabolizable glucose analog; it is transported with the same transporters as glucose, but it cannot be further metabolized and is washed out from the body by the kidneys. A previous study on chemical exchange saturation transfer (CEST) MRI showed that OMG had a stronger and longer-lasting effect than glucose [51]. In addition, OMG has little to no effect on the hormone regulation of blood glucose levels, allowing for higher blood concentrations than regular glucose [52]. Hartmann et al*.* [53] synthesized deuterated OMG (3-O-C^2^H_3_-glucose) and performed in vivo DMI measurements to analyze the uptake and washout of OMG in a rat model of breast cancer bone metastases at 7 T. Compared with [6,6’-^2^H_2_]glucose, the ^2^H resonance of deuterated OMG (at 3.5 ppm) is better separated from the HDO peak, and the HDO and OMG peaks were clearly distinguishable in vivo, with a peak OMG concentration at 3 min after intravenous injection. However, in-depth analysis of tumor tissue versus healthy tissue could not be performed due to the inhomogeneous transmit profile and the small sensitive area of the surface coil.

#### Pyruvate

Pyruvate is the end product of glucose metabolism through glycolysis. Since hyperpolarized ^13^C-MRS studies of tumor metabolism mostly use hyperpolarized [1-^13^C]pyruvate as substrate, Montrazi et al. [54] were inspired to investigate the use of [3,3’,3”-^2^H_3_]pyruvate for DMI and compare it with [6,6’-^2^H_2_]glucose. Directly after bolus injection of [3,3’,3”- ^2^H_3_]pyruvate in a pancreatic cancer mouse model, ^2^H-pyruvate and ^2^H-lactate signals became visible, but they faded very rapidly already ~5 min post-injection. The lactate signal was visible throughout the whole body and had only a weak sensitivity for the tumor, with a slightly higher concentration at the tumor rim. In contrast, ^2^H-lactate production after a bolus injection of [6,6’-^2^H_2_]glucose was clearly localized within the tumor, and the tumor ^2^H-lactate signal kept increasing for more than an hour post-injection. The ^2^H-glucose signal itself was mostly localized within the tumor rim, which could be explained by the high rate of glucose-to-lactate conversion inside the tumor. From this study, it was concluded that using glucose for the detection of pancreatic tumors with DMI is superior compared to pyruvate.

#### Fructose

Zhang et al. [55] tested the feasibility of using DMI with [6,6’-^2^H_2_]fructose to detect and localize tumors in a mouse model with subcutaneously implanted liver cancer cells and compared it with [6,6’-^2^H_2_]glucose. Lactate production rate was about two-fold lower for fructose than for glucose, and lactate signals were only detectable after summing the individual spectra over a 30 min time course. The HDO production rate was comparable for fructose and glucose, and it was proposed that HDO may serve as a sensitive indicator of substrate utilization in liver tumors. However, when using glucose, there is more wash in from HDO produced in other organs, whereas the HDO produced from fructose is more likely to reflect metabolism within the liver tumor itself, providing improved specificity.

#### Choline

Veltien et al. [49] investigated whether DMI can be utilized to measure the uptake and conversion of [^2^H_9_]choline in subcutaneously grown human renal carcinoma cells in mice. The [^2^H_9_]choline peak could clearly be identified in the acquired spectra, and choline was found to be heterogeneously distributed throughout the tumors. Following infusion, the [^2^H_9_]choline signal peaked rapidly and then decayed only slowly, while gradually broadening, indicating conversion into other choline-containing metabolites. In addition, the study demonstrated that [^2^H_9_]choline and [6,6’-^2^H_2_]glucose can be administered simultaneously, with their respective signals clearly distinguishable. This combined approach could enable more comprehensive metabolic characterization of tumors by simultaneously probing choline and glucose metabolism.

#### Fumarate

Studies exploiting [2,3-^2^H_2_]fumarate in combination with DMI to assess tumor necrosis upon treatment have been performed by Hesse et al. [56–58]. First, they investigated the feasibility of using [2,3-^2^H_2_]fumarate and ^2^H-MRS as an alternative to previous ^13^C-MR studies employing hyperpolarized [1,4-^13^C_2_]fumarate [56]. They examined mouse models of lymphoma, colorectal carcinoma, and breast cancer with a dose of 1 g/kg body weight [2,3-^2^H_2_]fumarate, administered intravenously, both before and after drug treatment at 7 T. For all three tumor types, a significant increase in [2,3-^2^H_2_]malate concentration was measured post-treatment compared with pre-treatment. Although the labeled fumarate concentrations increased as well, the malate/fumarate ratio increased significantly after drug treatment. Since the resonances of fumarate (at 6.5 ppm) and malate (at 2.4 ppm) are well separated from HDO, the method should also be applicable at clinical field strengths and follow-up studies, therefore, focused on clinical translation.

Oral administration of [2,3-^2^H_2_]fumarate was evaluated and compared with the outcomes obtained following intravenous administration [57]. Oral dosing offers advantages, such as lower costs and increased patient comfort. In the treated murine lymphoma model, the maximum [2,3-^2^H_2_]malate signal detected within 65 min after [2,3-^2^H_2_]fumarate administration showed comparable SNR for oral and intravenous delivery. The increase in malate/fumarate ratio post-treatment was slightly reduced using oral administration compared with the intravenous method, yet the two methods yielded largely comparable results.

Another study explored the limits of the sensitivity of the method by lowering the concentration of intravenously administered [2,3-^2^H_2_]fumarate and by lowering the drug dose in the murine breast cancer model to induce differences in the extent of tumor cell death [58]. Even with the lowest drug dose, a measurable malate signal was obtained. The malate/fumarate ratio plateaued at tumor fumarate concentrations of 2 mM, which was achieved with administration of 0.3 g/kg [2,3-^2^H_2_]fumarate or more. The percentage of dead cells, as determined by histological assessment, was linearly correlated with the malate/fumarate ratio, indicating that this ratio provides a sensitive estimation of tumor cell death.

Together, these studies support the feasibility of using nontoxic fumarate concentrations, combined with oral administration, to enable translation of the method to clinical applications [57, 58].

#### ^2^H_2_O

Brender et al*.* [59] and Asano et al*.* [60] demonstrated that systemic administration of ^2^H_2_O combined with in vivo ^2^H-MR enables noninvasive visualization of tumor tissue based on deuterium accumulation. In both studies, the highest ^2^H signal was observed in tumor tissue compared to surrounding healthy tissue. Brender et al*.* [59] showed that a one-week ^2^H_2_O labeling period produced strong tumor contrast in colorectal and pancreatic cancer models at 9.4 T, and Asano et al*.* [60] confirmed these findings at a clinical field strength of 1.5 T, observing clear tumor-specific labeling already after one day of ^2^H_2_O administration. Furthermore, Asano et al*.* [60] demonstrated that mice treated with radiotherapy or chemotherapy showed a reduction in the tumor ^2^H signal already from day 1 of the treatment, preceding any visible morphological changes. Treatment efficacy was later confirmed by either tumor shrinkage or histological evidence of cellular apoptosis, underscoring the translational potential for therapy monitoring in clinical oncology. However, the underlying mechanisms responsible for the increased ^2^H labeling in tumor tissue compared with healthy tissue upon administration of ^2^H_2_O still need to be elucidated.

### Other organs and tissues

### Pancreas

In clinical practice, it has proven challenging to distinguish pancreatic cancer and pancreatitis, since they often present with similar clinical symptoms and radiologic characteristics [61, 62]. Inspired by DMI studies showing the Warburg effect in pancreatic cancer models [48, 63], Montrazi et al. [64] investigated whether DMI can be used to differentiate between pancreatic cancer and pancreatitis. They administered [6,6’-^2^H_2_]glucose intravenously in mice with pancreatic tumors or cerulein-induced acute pancreatitis and performed DMI at 15.2 T. Clear ^2^H-lactate signals were detected in the pancreatic tumors, while no ^2^H-lactate signals were detected in acute pancreatitis, indicating that DMI can distinguish between these conditions. Applicability at a lower field strength of 7 T was suggested, whereas translation to clinical field strengths was deemed unfeasible, due to reduced sensitivity and the resulting long scan times.

### Kidney

**Fig. 3:**
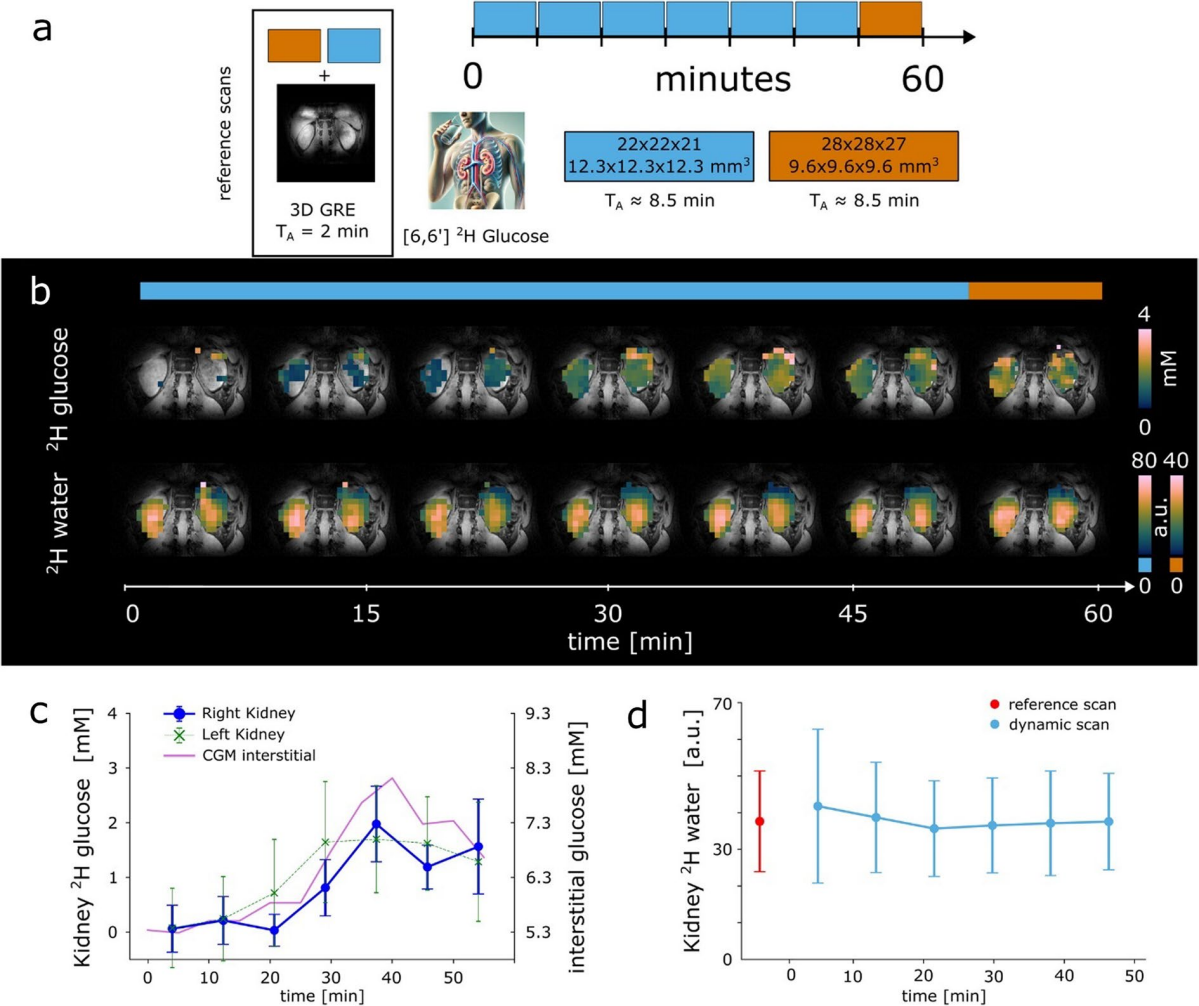
Dynamic 3D DMI data acquired of the human kidneys at baseline and 0–60 min after oral intake of [6,6’-^2^H_2_]glucose. (**A**) Overview of the scan protocol. (**B**) Metabolic maps of ^2^H-glucose and HDO overlaid on anatomical coronal images. (**C**) Time courses of ^2^H-glucose concentrations in the left and right kidney, together with total glucose concentrations in interstitial tissue measured using a continuous glucose monitoring sensor on the upper arm. (**D**) Time course of HDO concentration averaged across both kidneys. This figure was reproduced from Niess et al. [65] under the Creative Commons CC BY license

Renal DMI may uncover metabolic alterations underlying kidney dysfunction in diseases, such as diabetes and chronic kidney disease. Two studies at 7 T demonstrated the feasibility of dynamically measuring renal [6,6’-^2^H_2_]glucose uptake in healthy human subjects, revealing that glucose dynamics in the kidney closely resemble that in the liver [25] and interstitial tissue [65]. Niess et al. [65] further demonstrated the feasibility of assessing renal ^2^H_2_O uptake and distribution in vivo (Fig. 3). McLean et al. [66] applied this approach at 3 T in a patient with a benign renal tumor (oncocytoma), observing higher HDO signals in the tumor compared with adjacent normal-appearing kidney tissue. Although these results require validation in larger cohorts, they support the potential of DMI as a noninvasive method for studying renal pathophysiology.

Fries et al*.* [67] introduced the concept of using deuterium-labeled nanoparticles, specifically poly(amidoamine) (PAMAM) dendrimers with deuterated acetyl groups at the surface (PAMAM-G5-[^2^H_3_]Ac), as DMI contrast agents. By tracking PAMAM-G5-[^2^H_3_]Ac in mice, they visualized renal uptake and clearance. The high-resolution images acquired at 15.2 T showed substructures within the kidney, with higher PAMAM-G5-[^2^H_3_]Ac concentrations in the medulla. Since nanoparticles such as PAMAM dendrimers can be functionalized for targeted drug delivery, this approach offers a promising means to noninvasively monitor the biodistribution and kinetics of therapeutic particles using DMI.

### Heart

Cardiac metabolism is unique compared to that of other organs and muscles in the body. It has a continuous high energy demand, large mitochondrial density, and under healthy conditions, it is metabolically omnivorous, deriving roughly 70% of its energy from fatty acids and 30% from glucose. In pathological hypertrophy, this balance shifts toward increased glucose utilization, and other heart diseases are also linked to abnormal myocardial energy metabolism [68]. DMI seems well-suited to study cardiac metabolism because of its ability to trace different deuterated substrates and their downstream metabolites. Using this approach at 16.4 T, Wang et al. [69] investigated myocardial metabolism in mice following intravenous administration of [2,2’,2”-^2^H_3_]acetate or [6,6’-^2^H_2_]glucose. Acetate infusion led to a six-fold higher HDO accumulation rate than glucose infusion, without correcting for the number of deuterons in the substrates. Additionally, in mice infused with acetate, a ^2^H-Glx peak was observed in the acquired spectra, which was negligible after glucose administration. Collectively, these results indicate that the healthy heart more efficiently uses acetate compared to glucose. Future studies using fatty acid substrates could explore whether DMI can detect shifts from fatty acid to glucose metabolism in pathological conditions.

### Skeletal muscle

Deuterium has a quadrupolar moment, which can lead to peak broadening or even splitting when molecular motion is not fully isotropic, as is the case in spatially ordered tissues. This effect was previously observed *ex vivo* in tendons [70], and more recently, Gursan et al. [71] demonstrated it in vivo for the naturally abundant HDO signal in lower-leg skeletal muscles. They showed that the residual quadrupolar coupling of the HDO signal depends on the angle (θ) between the muscle fibers and the main magnetic field, following a 3*cos*^2^θ – 1 relationship. Similar residual quadrupolar couplings are expected for other ^2^H signals in skeletal muscle, and should therefore be considered in future DMI studies with ^2^H-labeled substrates in skeletal muscle or other spatially organized tissues.

### Fetoplacental tissue

Preeclampsia is a pregnancy-related disorder characterized by maternal high blood pressure and placental hypoxia, leading to increased anaerobic metabolism in placental tissue and potential fetal organ damage [72]. Current pregnancy examinations using ultrasound or MRI are mainly focused on morphology, limiting the ability to assess fetoplacental metabolism. Moreover, safety precautions restrict the range of imaging techniques that can be applied during pregnancy. DMI potentially provides a safe, non-invasive method to analyze fetoplacental energy metabolism, particularly in preeclamptic conditions. Markovic et al. [73] induced preeclampsia in pregnant mice using a vasoconstrictor and compared them with healthy controls. DMI data was collected from the abdomen of the mice at 15.2 T upon intravenous infusion of [6,6’-^2^H_2_]glucose. Increased ^2^H-glucose, HDO, and especially ^2^H-lactate concentrations were observed in the fetuses and placenta of the preeclamptic mice compared with the healthy controls, showing the potential of DMI to characterize disturbances in fetoplacental metabolism in preeclampsia.

### Brown adipose tissue

Activated brown adipose tissue (BAT) is associated with increased energy expenditure, making it of interest for therapies targeting metabolic syndrome and obesity. Activation of BAT can be induced by, for example, cold exposure [74]. Earlier [^18^F]fluoro-2-deoxyglucose positron emission tomography (^18^F-FDG-PET) studies have shown increased glucose uptake in activated BAT compared with non-activated BAT [75, 76]. However, downsides of ^18^F-FDG-PET are the associated radiation exposure and the lack of information on downstream metabolites of glucose. DMI could potentially overcome both of these disadvantages, and thus Riis-Vestergaard et al. [77] evaluated whether DMI could be used to investigate glucose metabolism in BAT of cold-acclimatized rats versus a thermoneutral control group. After one week of housing in a cold or thermoneutral environment, [6,6’-^2^H_2_]glucose was injected intravenously, and the rats were scanned at 9.4 T. DMI successfully distinguished differences in glucose metabolism between the two groups, revealing a significantly larger glucose uptake in the cold-acclimatized rats. Additionally, BAT of cold-acclimatized rats showed higher HDO and lower ^2^H-Glx levels, suggesting a shift toward anaerobic glycolysis, although no significant difference in ^2^H-lactate was observed.

### Inflammation

Metabolism plays an important role during inflammatory responses. To meet the increased energy demands required for activation and proliferation, immune cells primarily rely on anaerobic glycolysis [78]. Additionally, inflammatory cytokines can disturb glucose and lipid metabolism and induce insulin resistance [79]. Consequently, chronic low-grade inflammation is closely linked to several metabolic diseases, such as obesity, diabetes, and cardiovascular disease [80].

Flocke et al*.* [81] showed that alterations in glucose metabolism in inflammation can be quantified using DMI. Plugs containing either endotoxin (lipopolysaccharide (LPS)) or a control solution were implanted in the necks of mice, and DMI scans were acquired before and for 60 min after intraperitoneal [6,6’-^2^H_2_]glucose injection. As expected, the LPS-induced inflammatory sites showed enhanced anaerobic glycolysis, reflected by a significantly faster ^2^H-glucose decline and increased ^2^H-lactate production compared with the controls. Inflammatory sites also showed a smaller initial rise in HDO, consistent with reduced oxidative metabolism through the TCA cycle, where the ^2^H label can be transferred to water in multiple steps. To complement these metabolic measurements, the authors tracked ^19^F-labeled immune cells using ^19^F-MRI, which confirmed immune cell infiltration at the LPS sites but not in controls. The combination of DMI and ^19^F-MRI, therefore, seems a promising strategy to gain more insight into the interplay between metabolism and inflammatory responses.

In addition to renal imaging, Fries et al*.* [67] demonstrated that deuterium-labeled PAMAM-G5-[^2^H_3_]Ac nanoparticles can also be used to visualize inflammation. In this study, a local inflammatory response was induced in the right hind leg of mice, and PAMAM-G5-[^2^H_3_]Ac was injected subcutaneously into both footpads. High-resolution ^2^H-MR images revealed accumulation of the nanoparticles in the major lymph nodes of the inflamed leg, whereas no uptake was observed on the contralateral healthy side. These findings highlight the potential of deuterium-labeled nanoparticles as DMI tracers for detecting and monitoring inflammatory activity in vivo.

## Technical advancements

This paragraph highlights technical advances in DMI for body applications. A broader and more comprehensive overview of methodological developments is provided in the dedicated review by Niess et al. [82] in this special issue.

### Radiofrequency coils

To be able to perform DMI experiments, specific hardware is required, including RF coils tuned to the ^2^H frequency. For DMI studies of the body, both in animal models and in humans, ^2^H surface coils are most commonly used [8, 29, 47, 66, 69, 77]. However, surface coils suffer from inhomogeneous B_1_ fields and limited penetration depth. To overcome this limitation, some preclinical studies have used combinations of ^2^H and ^1^H volume coils, allowing convenient acquisition of ^1^H-MRI scans for anatomical reference and B_0_ shimming, while also enabling metabolic ^2^H-MRS measurements [8, 59, 60, 83]. Volume coils provide large coverage, suitable for whole-animal imaging, and a homogeneous B_1_ field. However, their sensitivity is inherently low. Therefore, an optimal setup would use the volume coil only for ^2^H RF excitation, taking advantage of its transmission uniformity, combined with a ^2^H surface coil array for signal reception to maximize SNR [83].

To enable DMI across the human abdomen at 7 T, Gursan et al. [25] developed a body array coil consisting of anterior and posterior elements, each containing two ^2^H transmit/receive loops for DMI and two ^1^H dipole antennas for anatomical imaging and B_0_ shimming. Simulated and measured B_1_^+^ patterns showed good correspondence, and the coil provided sufficient sensitivity and coverage for DMI in liver, stomach, and right kidney although flip-angle non-uniformity remained a limitation [25]. Expanding on this work, Gursan et al. [84] presented a double-tuned ^2^H/^31^P whole-body birdcage transmit coil integrated into the outer structure of the patient tube of a whole-body 7 T MRI scanner. This design offers homogeneous B_1_^+^ fields across large anatomical regions, and, in combination with a 8-channel ^2^H/^31^P body receive array, provides high sensitivity for combined ^2^H- and ^31^P-MRS throughout the body, without the need for coil repositioning. Together, these developments mark important progress toward robust, whole-body DMI in humans.

While DMI may be regarded as a substitute for ^13^C-MRS, Poli et al*.* [26] worked to integrate the two methods using a triple-tuned ^1^H/^2^H/^13^C surface coil. This coil enabled interleaved measurements of liver glucose uptake with DMI, and liver glycogen storage, not detectable by ^2^H-MRS, with ^13^C-MRS. In this manner, the triple-tuned ^1^H/^2^H/^13^C coil allowed complementary metabolic information to be acquired under identical physiological conditions, although measuring changes in ^13^C-glycogen levels proved challenging.

### Pulse sequences

**Fig. 4:**
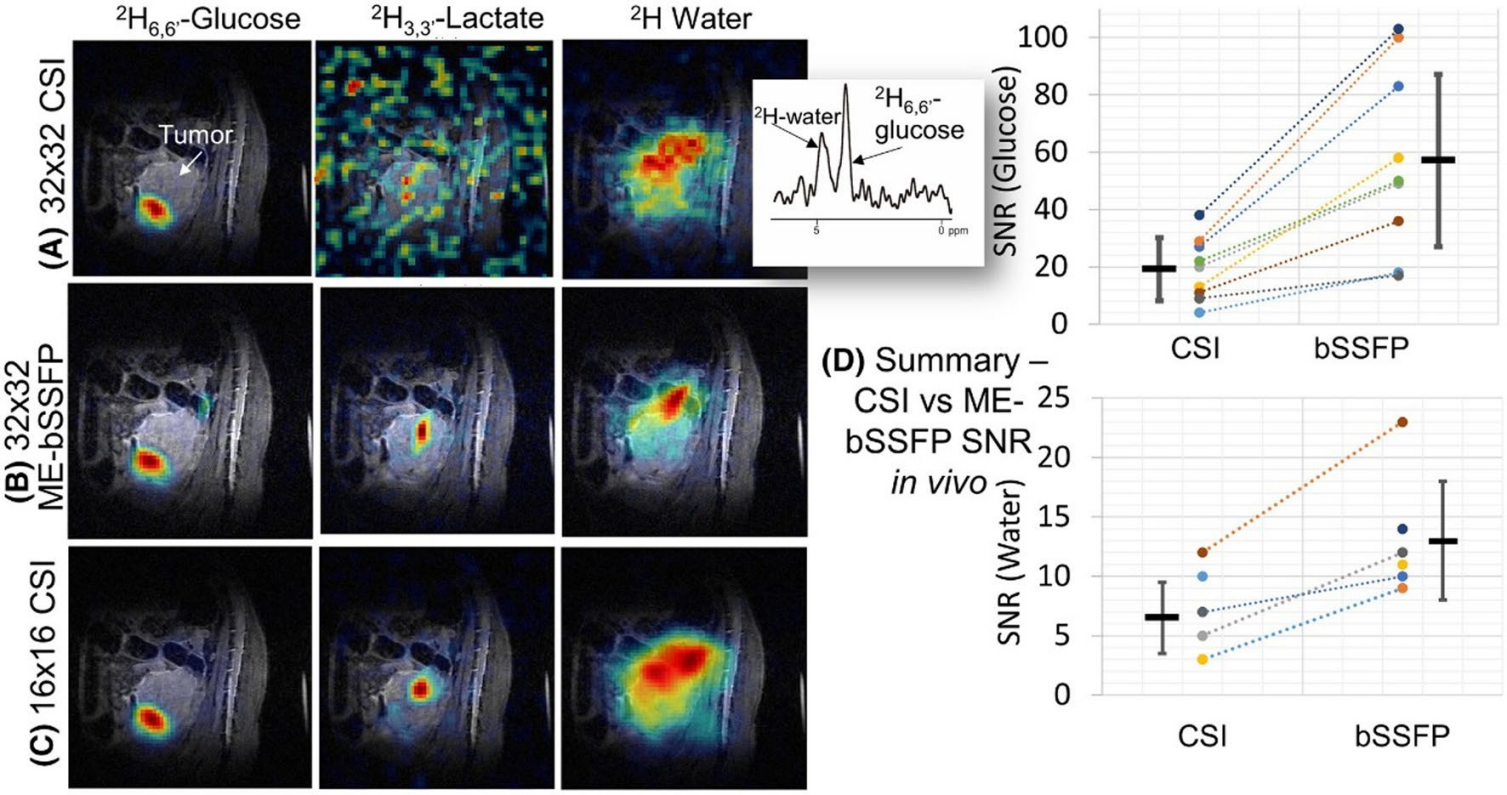
2D DMI data acquired of a mouse model of pancreatic adenocarcinoma after injection of [6,6’-^2^H_2_]glucose. (**A**) ^2^H-glucose, ^2^H-lactate, and HDO maps acquired with MRSI (32 × 32 matrix size), referred to as chemical shift imaging (CSI) here, overlaid on anatomical images with the tumor indicated. Lactate is not distinguishable as illustrated in the shown spectrum. (**B**) ^2^H-glucose, ^2^H-lactate, and HDO maps acquired with ME-bSSFP (32 × 32 matrix size). (**C**) ^2^H-glucose, ^2^H-lactate, and HDO maps acquired with CSI with a lower spatial resolution (16 × 16 matrix size) in order to enable lactate detection. (**D**) SNR values of ^2^H-glucose and HDO for the CSI and ME-bSSFP scans (both 32 × 32 matrix size). On average, the SNR of ^2^H-glucose is three-fold higher and the SNR of HDO is two-fold higher for ME-bSSFP compared to CSI. This figure was reproduced from Peters et al. [63] with permission

DMI data are most often acquired using 2D or 3D phase-encoded MRSI. In this approach, only one point in k-space is acquired per repetition time, leading to slow measurements and thereby limiting temporal and/or spatial resolution. To accelerate spatial encoding and improve spatial resolution, Peters et al. [63] developed a multi-echo balanced steady-state free precession (ME-bSSFP) sequence. ME-bSSFP yielded a two- to three-fold improved SNR compared to conventional MRSI for DMI in a mouse model of pancreatic adenocarcinoma (Fig  4.). The Iterative Decomposition of Water and Fat with Echo Asymmetry and Least-Squares Estimation (IDEAL) framework was used to resolve the different metabolites, i.e., ^2^H-glucose, ^2^H-lactate and HDO. Time courses of these metabolites did not differ significantly between the two acquisition methods, but the localization of glucose and lactate was more clearer in the ME-bSSFP data, which was acquired with the same temporal resolution as the MRSI data, but with a higher in-plane spatial resolution (1.3 x 1.3 mm^2^ versus 5 x 5 mm^2^).

Disadvantages of ME-bSSFP include the use of fly-back gradients, which reduce the efficiency of the pulse sequence. Additionally, weighted averaging cannot be applied in the echo dimension. To overcome these challenges, Montrazi et al*.* [64] developed a 2D Hanning-weighted MRSI-SSFP sequence, providing an additional two-fold SNR gain with respect to ME-bSSFP.

Nam et al*.* [85] showed that a significant increase in temporal and/or spatial resolution can be achieved using ^2^H echo-planar spectroscopic imaging (EPSI). SNR per unit time was slightly lower using EPSI compared to conventional MRSI due to the absence of data sampling during the gradient ramps. However, using EPSI, liver DMI data could be acquired over a large field of view (360 × 240 × 300 mm^3^) with a nominal voxel size of 20 mm isotropic in just 2 min, while maintaining sufficient SNR to resolve both HDO and ^2^H-glucose peaks after administration of [6,6’-^2^H_2_]glucose. In comparison, to cover the same field of view, a phase-encoded MRSI scan with the same spatial resolution would take at least 20 min for a single average.

Based on previous work demonstrating enhanced spatial resolution for dynamic DMI in the brain using a concentric ring trajectory readout [86], Niess et al*.* [65] applied the same approach to the kidneys. They achieved sufficient SNR to quantify ^2^H-glucose and HDO with voxel volumes of 0.9–1.8 mL and a temporal resolution of 8.5 min although they were unable to detect regional differences between the cortex and the medulla. Without averaging, the concentric ring trajectory readout would allow a minimum scan duration of 1–2 min for the used matrix size, creating flexibility to improve spatial and/or temporal resolution even further when SNR is not limiting.

### Reconstruction

To further enhance the SNR gain as provided by the ME-bSSFP sequence [63], Montrazi et al. [87] refined the IDEAL spectral reconstruction, making use of the time-dependency of the metabolite signals in dynamic data. They introduced two reconstruction strategies: one imposing regularization across the kinetic series (RK‑SpecRecon), and another using subspace-constrained kinetic representations co-processed with the spectral dimension (SK‑SpecRecon). Both reconstruction methods delivered a two- to three-fold SNR gain without loss of metabolite‑specific information. Additionally, different denoising methods for the resulting ^2^H images were evaluated, showing that compressed‑sensing multiplicative denoising [88] achieved the greatest SNR improvement albeit at higher computational cost than block‑matching/3D filtering [89], which still outperformed standard apodization [87].

Nam et al. [90] implemented a low-rank and subspace model-based reconstruction framework, a method that exploits spatio-spectral correlation. This approach provided higher SNR and lower Cramer–Rao lower bounds compared with regular Fourier transform-based reconstruction of in vivo ^2^H data acquired with a conventional MRSI sequence.

The SNR gains from these advanced reconstruction and denoising methods can be leveraged to improve temporal or spatial resolution, enabling acquisition parameters to be optimized according to the specific goals of a study.

### Motion

Body MR imaging and spectroscopy are strongly affected by motion arising from respiration, cardiac pulsation, and peristalsis. For example, the liver can be displaced by 7.5 cm during breathing [91]. The main challenges associated with motion are localization errors and local B_0_ field variations. Localization errors cause signal misattribution and increased partial volume effects, while varying B_0_ fields broaden spectral lines and degrade spectral quality, making it more difficult to resolve neighboring peaks [92]. Although no studies have yet addressed motion correction specifically for ^2^H-MRSI of the body, Wampl et al. [93] developed a flexible, ^1^H-MR navigator-based framework for motion corrected X-nuclei imaging, potentially applicable to ^2^H as well. Their method enables prospective and retrospective correction in up to three directions using Open Source Computer Vision Library (OpenCV) object tracking, and they demonstrated improved localization and reduced signal bleed in ^31^P-MRSI of the heart at 7 T.

## Outlook and conclusions

This review has summarized the current state of in vivo DMI research in body applications. Over the past seven years, DMI has been applied across a broad range of studies, and its scope continues to expand as new ^2^H-labeled substrates, organs, and pathologies are investigated.

 How does DMI compare with other metabolic imaging techniques, and what additional information can it provide? As mentioned in the Introduction, ^13^C-MRS has traditionally been used for tracking metabolic pathways in vivo with MR, but its low intrinsic sensitivity limits spatial resolution. Hyperpolarization can increase the sensitivity of ^13^C-MR by more than 10,000-fold, enabling spatial mapping [94]. However, the enhanced polarization decays with the T_1_ relaxation time, typically limiting the usable signal to only 1–2 min. Hence, hyperpolarized ^13^C-MR is only suitable for imaging rapid processes, whereas with DMI also slower metabolic pathways can be assessed. Moreover, hyperpolarization is technically complex and costly, requiring specialized hardware.

Another commonly used method for metabolic imaging is nuclear imaging. ^18^FDG-PET, which is specific for glucose imaging, is widely used in the clinic for tumor staging, detecting recurrence, and assessing therapy response. A disadvantage of PET imaging is the use of ionizing radiation. Additionally, ^18^FDG gets trapped in cells after phosphorylation, allowing measurement of glucose uptake but preventing assessment of downstream metabolites and metabolic fluxes [95]. By contrast, DMI with deuterated glucose can track both uptake and subsequent metabolic transformations, providing complementary information to PET.

Like ^13^C-MR and FDG-PET, DMI also faces several challenges, some of which have already been discussed in previous paragraphs. Most importantly, ^2^H-MRSI is relatively insensitive and its spatial and temporal resolution remain below that of conventional ^1^H-MRI. Both are crucial for clinical translation: spatial resolution is needed to resolve small structures or local metabolic heterogeneity, whereas temporal resolution ensures clinically acceptable scan times that remain tolerable for patients, especially those with severe conditions. To date, most DMI studies have used ultra-high field MR scanners to increase sensitivity. While this has provided valuable research data, it limits clinical applicability. Studies at clinical field strengths are necessary to assess how the reduced sensitivity, and concomitant compromises in spatial and/or temporal resolution, impact the applicability of DMI in a clinical setting. In addition, wider availability of clinical scanners equipped for ^2^H imaging is essential for translating this technique into routine clinical practice. Currently, ^2^H RF coils are often custom-made, highlighting the need for more standardized hardware solutions.

Improving the spatial and temporal resolution of DMI requires advances in both data acquisition and processing. On the acquisition side, the use of ^2^H volume transmit coils combined with multichannel receive arrays could enhance imaging of deeper body regions. In addition, new pulse sequences have shown the potential to accelerate data acquisition and reduce scan times. On the processing side, new denoising and reconstruction methods can enhance SNR independently of acquisition methods. In this area, innovations developed for cerebral ^2^H-MRSI are often directly transferable to body applications. However, motion-related challenges, particularly those arising from respiratory and cardiac motion, remain largely specific to body applications and represent a nearly unexplored area.

Most of the studies on body applications of DMI to date have been preclinical, conducted primarily in small animals. Such studies benefit from controlled conditions, including the ability to anesthetize animals to reduce motion and accommodate longer scan times. Importantly, apart from deuterated glucose and ^2^H_2_O, all other deuterated substrates described in this review have so far only been applied in animal models, and their translation to humans remains to be established. Similarly, while DMI in the liver and kidney has been demonstrated in humans, applications in other body organs remain largely preclinical.

The clinical feasibility of ^2^H-MRSI for body applications has been implied by several studies discussed in this review, but further development is needed. Although various potential use cases have been identified, the technique has yet to demonstrate clear added value for clinical decision making, whether for diagnosis or therapy monitoring. Larger in vivo human studies will be crucial to assess repeatability, reproducibility, and robustness, and to support the development of standardized protocols for body imaging. If these technical and methodological developments continue, ^2^H-MRSI could become a valuable tool for metabolic imaging of body organs in clinical practice.

## Abbreviations

^13^C: Carbon-13
^18^F-FDG-PET: [^18^F]fluoro-2-deoxyglucose positron emission tomography
^2^H: Deuterium
Ac: Acetyl
BAT: Brown adipose tissue
CEST: Chemical exchange saturation transfer
CSI: Chemical shift imaging
DMI: Deuterium metabolic imaging
DNL: De novo lipogenesis
EPSI: Echo-planar spectroscopic imaging
FLASH: Fast low-angle single shot
Glx: Combined glutamate + glutamine
IDEAL: Iterative decomposition of water and fat with echo asymmetry and least-squares estimation
LPS: Lipopolysaccharide
MASLD: Metabolic dysfunction-associated steatotic liver disease
ME-bSSFP: Multi-echo balanced steady-state free precession
MR: Magnetic resonance
MRI: Magnetic resonance imaging
MRS: Magnetic resonance spectroscopy
MRSI: Magnetic resonance spectroscopic imaging
NMR: Nuclear magnetic resonance
OMG: 3-O-Methylglucose
PAMAM: Poly-(amidoamine)
ppm: Parts per million
RF: Radiofrequency
RK-SpecRecon: Regularized kinetics spectral reconstruction
RYGB: Roux-en-Y gastric bypass
SK-SpecRecon: Subspace-constrained kinetic spectral reconstruction
SNR: Signal-to-noise ratio
T: Tesla
TCA: Tricarboxylic acid
TMA: Trimethylamine
TMAO: Trimethylamine *N*-oxide
